# Prevalence and Characteristics of Novel Pathogenic *Leptospira* Species in Bats in Yunnan Province, China

**DOI:** 10.3390/microorganisms11061619

**Published:** 2023-06-20

**Authors:** Tian Yang, Weihong Yang, Guopeng Kuang, Hong Pan, Xi Han, Lifen Yang, Juan Wang, Yun Feng

**Affiliations:** 1 School of Public Health, Dali University, Dali 671000, China; 2 Yunnan Provincial Key Laboratory for Zoonosis Control and Prevention, Yunnan Institute of Endemic Disease Control and Prevention, Dali 671000, China

**Keywords:** leptospirosis, pathogenic *Leptospira*, bats, zoonosis, Yunnan province

## Abstract

Leptospirosis has been identified as a zoonotic disease caused by pathogenic spirochetes of the bacterial genus *Leptospira*. Rodents are considered the primary hosts of these bacteria, whereas many recent studies suggest that bats may serve as potential natural reservoirs. However, studies on pathogenic spirochetes hosted by bat populations still need to be completed in China. In this study, a total of 276 bats belonging to five genera collected in Yunnan Province (Southwest China) from 2017 to 2021 were included in the screening. Pathogenic spirochetes were detected by PCR amplification and sequencing targeting four genes (*rrs*, *secY*, *flaB,* and *LipL32*), resulting in 17 positive samples. Phylogenetic analysis based on multi-loci concatenated sequences, inferred by MLST approach, identified the strains as two novel *Leptospira* species within the pathogenic group. Of note, only *Rousettus leschenaultii* was found to harbor these spirochetes, suggesting it may be one of the potential natural reservoirs in circulating leptospires in this region. Nevertheless, the pathogenesis and transmission dynamics still need to be fully understood, requiring in-depth studies on other animals and the surrounding population.

## 1. Introduction

Leptospirosis, a re-emerging disease caused by infection with pathogenic spirochetes of the genus *Leptospira*, is estimated to annually affect more than 1 million people resulting in approximately 60,000 deaths [1,2,3,4]. The disease is a worldwide infection with a much greater incidence in tropical and subtropical countries, and China is one of the major endemic areas [5,6,7]. The incidence of leptospirosis varies among different provinces in China, with an overall incidence of 2.5018/100,000 in Yunnan Province between 2007 and 2018 [8]. Currently, at least 64 species and 250 serovars of *Leptospira* have been recognized [5,9,10]. Based on pathogenicity and genetic clustering, the genus can be divided into three lineages—pathogenic, intermediate, and saprophytic groups [1,6,11]. Among these, *L. interrogans*, *L. borgpetersenii*, and *L. kirschneri*, which cluster into the pathogenic group, are the main causative agents of leptospirosis for zoonotic infection [12,13]. Besides, new species within the pathogenic group have been discovered in soil and water; however, their pathogenicity remains unclear [9,14,15].

The role of small mammals as the most critical reservoir hosts and vectors for transmitting leptospirosis cannot be ignored [2,6,7]. To date, more than 200 species of animals have been identified as hosts of leptospires worldwide, including mammals such as rats, dogs, cattle, pigs, horses, and sheep [1,16]. Of these, rats are considered the primary reservoirs of pathogenic *Leptospira* [2,6]. However, *Leptospira* spp. has been detected in over 50 species of the order *Chiroptera* in tropical and subtropical regions and parts of Europe, suggesting that bats can be the natural reservoirs of these zoonotic microorganisms [17,18,19,20,21,22,23,24,25,26]. In China, several studies have corroborated that bats harbored these bacteria [27,28]. However, little is known about the maintenance and transmission of pathogenic leptospires among bats, and their correlation with human leptospirosis.

Leptospires can enter through mucous membranes or open skin wounds and then migrate rapidly to tissues and organs within their hosts, including livers, lungs, and kidneys [2,29,30]. The pathogens are usually colonized in the kidneys of infected mammals, which may cause them to excrete with host urine for days or even months [29,30,31,32]. Humans and animals are mostly infected through direct contact with the excretion of infected animals or the leptospires-contaminated environment [5,6]. The clinical manifestations of leptospirosis vary in severity and complexity, with symptoms such as diffuse pulmonary hemorrhage, jaundice, and renal failure occurring with prolonged infection [4,6,33,34].

*Leptospira* spp. can be identified at the species level by phylogenetic analysis of the initial region of the 16S ribosomal gene (*rrs*); however, it is impossible to distinguish some species by this gene [35]. Indeed, Multilocus sequence typing (MLST) is the choice for genotyping many bacterial pathogens (including *Leptospira*) because of its well-inferred evolutionary history that can be more accurate than single-locus analyses [36,37,38]. Previously, house-keeping genes such as preprotein convertase gene (*secY*), outer membrane lipoprotein gene 32 (*LipL32*), and outer membrane lipoprotein gene 41 (*LipL41*), were selected to compose the MLST schemes for inferring the genetic clustering of pathogenic *Leptospira* species, especially *L. interrogans*, *L. noguchii,* and *L. kirschneri* [36,39,40]. The multiple advantages of generating sequences from different genes at a high throughput scale allow for defining the novel species effectively; besides, they are suitable for studies regarding both intra-species and inter-species relationships of the genus [36].

Yunnan province in southwestern China is characterized by high levels of bat biodiversity [41]. Recently, a large number of pathogens, including some zoonotic viruses and bacteria with public health threats, have been identified in bats in Yunnan [42,43]. To date, however, studies on pathogenic spirochetes in bat populations are limited. Accordingly, we investigated the pathogenic *Leptospira* carried by bats collected in Yunnan, aiming to provide a more solid understanding of leptospirosis in bats and public health issues.

## 2. Materials and Methods

### 2.1. Ethics Statement 

The protocols for sampling and handling used in this study were reviewed and approved by the Medical Ethics Committee of the Yunnan Institute of Endemic Disease Control (File No. 20160002). The Yunnan Institute of Endemic Disease Control and Prevention Biosafety Committee approved all experiments. All experimental operations were performed in biosafety cabinets in compliance with biosafety regulations.

### 2.2. Bat Sample Collection 

**Figure 1:**
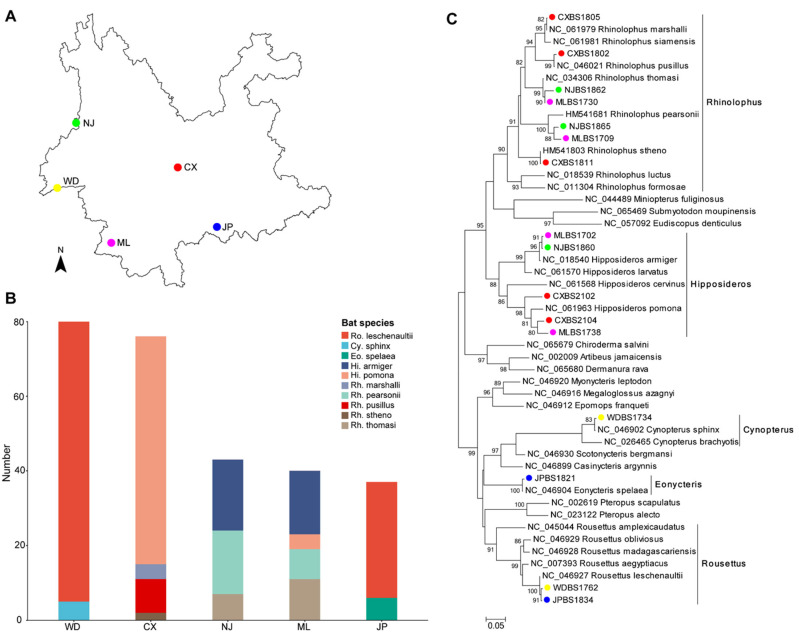
Overview of geographic distribution and species identification of bats in this study. (**A**) Geographic distribution of bats in Yunnan Province. Different colors indicate bats collected from five sampling sites, including CX (red), JP (blue), ML (purple), WD (yellow), and NJ (green); (**B**) Bat species contribution of five sampling sites. Each color represents a different bat species, and the bar chart shows the species composition and abundance of bats; (**C**) Phylogenetic tree of bats. The tree was reconstructed based on the partial nucleotide sequences (658 bp) of the bat *COI* gene using the Maximum-likelihood (ML) method. Different colors indicate bats collected from five sampling sites and the same as (**A**).

Five sites in Yunnan Province were selected for sampling, labeled WD, ML, CX, NJ, and JP (Figure 1A). Between 2017 and 2021, sticky nets were set up around the orchards and caves, and the bats were promptly removed after being stuck. The captured bats were initially identified based on morphological traits, and the location and date information was recorded. The bats were anesthetized with ether and dissected in a biosafety cabinet. Kidney tissues were collected and placed in liquid nitrogen, transported back to the laboratory, and stored at −80 °C until further experiment.

### 2.3. Deoxyribonucleic Acid Extraction and Bats Species Confirmation

Total deoxyribonucleic acid (DNA) was extracted from individual kidney tissues using TIANamp Genomic DNA Kit (Tiangen Biotech, Beijing, China) and was processed following the instruction provided by the manufacturer. Species identification of bats was confirmed by analyzing the cytochrome c oxidase I (*COI*) gene. DNA extracted from the kidneys was used as a template, and the *COI* gene was amplified using the forward primer VF1d_t1 and reverse primer VR1d_t1 (Table S1) [44]. PCR reactions were under the following conditions: an initial cycle of 10 min at 94 °C, followed by 30 cycles of 94 °C for 30 s, 47 °C for 30 s, and 72 °C for 1 min, and the final extension at 72 °C for 10 min. PCR amplification was confirmed by electrophoresis on a 1.2% agarose gel. All PCR products were performed to Sanger sequencing, and species were identified based on obtained sequences compared with the BOLD and NCBI nucleotide database.

### 2.4. Detection of Pathogenic Leptospires

Total DNA extracted from bat kidney tissues was used as a template for screening for pathogenic *Leptospira* by nested PCR. In order to well characterize the detected pathogenic spirochetes, this study first amplified the gene *rrs* [45]. The initial screening was then confirmed by amplifying three other genes (all 276 samples were included in detection), including the genes *secY*, *LipL32*, and flagellin B gene (*flaB*) [46,47,48,49]. These genes are suitable for phylogenetic analysis of pathogenic *Leptospira* [47,48]. The sequences of each primer and related parameters are shown in Table S1.

The first round of nested PCR reactions was performed in a 25 μL mixture containing 12.5 μL 2 × DreamTaq Green PCR Master Mix (Thermo Scientific, Vilnius, Lithuania), 1 μL of 10 μM each forward and reverse primer (Sangon Biotech Co., Ltd., Shanghai, China), 8.5 μL nuclease-free water, and 2 μL sample DNA. PCR was performed with one denaturation cycle at 94 °C for 5 min; 40 amplification cycles at 94 °C for 30 s, 50 °C for 45 s, and 72 °C for 60 s; and an additional final extension at 72 °C for 10 min. The second round of PCR reactions were performed in a 50 μL mixture containing 25 μL of 2 × DreamTaq Green PCR Master Mix (Thermo Scientific, Vilnius, Lithuania), 1 μL of 10 μM each forward and reverse primers (Sangon Biotech Co. Ltd., Shanghai, China), 13 μL of nuclease-free water, and 4 μL of first-round reaction products per sample. After the PCR reaction, the second-round product was separated on 1.2% agarose gel electrophoresis and visualized with an E-Gel Imager (Tanon 2500B). The observed bands of the expected size were purified and sequenced by Sangon Biotech. The SeqMan program in the DNASTAR package (Lasergene) was used to confirm and assemble the sequences. The assembled sequences were compared with known sequences in GenBank using nucleotide BLAST (BLAST + 2.13.0) and identified based on homology.

### 2.5. Sequence Analysis

The representative sequences of all the genes mentioned above for known species of the genus *Leptospira* were searched and downloaded from GenBank (http://www.ncbi.nlm.nih.gov/genbank/, accessed on 17 December 2022). In order to identify the newly detected bacteria strains, the phylogenetic tree involving three major clusters of the genus—pathogenic group (n = 30), intermediate group (n = 12), and saprophytic group (n = 10)—was constructed based on *rrs* gene sequences (Table S2). *LipL32* is the major outer membrane protein usually found only in pathogenic *Leptospira* [50,51,52,53]. Therefore, the homology of nucleotide and amino acid sequences for this gene was selected for comparison with sequences of different species (n = 13) that were previously detected from humans by using the MegAlign program, which was implemented in the DNASTAR 7.1 software package (Lasergene). Subsequently, different parameters analyzed, including the number of alleles, the number of polymorphisms, and the discriminatory power (DP) of each locus (*LipL32*, *secY*, and *flaB*), were evaluated based on the complete sequences of 49 strains belonging to 16 pathogenic species by MLSTest, as well as concatenated loci. Sequence types (STs) of the concatenated sequences of novel strains were also assigned by MLSTest 1.0 software. The phylogenetic analysis with the 49 pathogenic strains was performed (2 strains of the intermediate group were included as outgroup) (Table S3). Briefly, MAFFT was used to align the nucleotide sequences [54], the terminal sequences were removed manually, and ambiguously aligned sequences were removed using trimAl [55]. Phylogenetic trees were reconstructed using the maximum likelihood method implemented in PhyML program, with GTR+G substitution model and SPR tree topology optimization algorithm [56].

## 3. Results

### 3.1. Collection and Species Identification of Bat

From 2017 to 2021, 276 bats were captured from five trapping sites at the border and central regions of Yunnan Province (Figure 1A). Initial identification of bats was based on morphological traits and further confirmed by phylogenetic analysis of the cytochrome c oxidase (*COI*) gene. As a result, the collected bats were identified as ten species residing in five genera (Figure 1C), including three frugivorous species (106 *Rousettus leschenaultii*, 6 *Eonycteris spelaea*, and 5 *Cynopterus sphinx*) and seven insectivorous species (65 *Hipposideros pomona*, 36 *Hipposideros armiger*, 25 *Rhinolophus pearsonii*, 18 *Rhinolophus thomasi*, 9 *Rhinolophus pusillus*, 4 *Rhinolophus marshalli*, and 2 *Rhinolophus stheno*) (Table S4). The number of bat species varied by disparate sampling sites, with two, four, three, four, and two species in sites WD, CX, NJ, ML, and JP, respectively (Figure 1B). Among these, *Rousettus leschenaultii* mainly collected in WD (27.17%) and JP (11.23%), while the others such as *Rhinolophus stheno* (0.72%), *Rhinolophus marshalli* (1.45%), and *Rhinolophus pusillus* (3.26%) were only captured in CX.

### 3.2. Detection and Identification of Leptospira 

All bat kidney tissues (n = 276) were first screened individually for pathogenic spirochetes with *rrs* gene fragment, resulting in 17 (6.16%) positive samples (Table S4). All spirochetes-positive samples were contributed by *Rousettus leschenaultii*. The subsequent confirmation by three additional genes fragment (*LipL32*, *secY*, and *flaB*) revealed the same result. Overall, four gene fragment sequences for 17 pathogenic *Leptospira* strains were successfully amplified and sequenced. 

**Figure 2:**
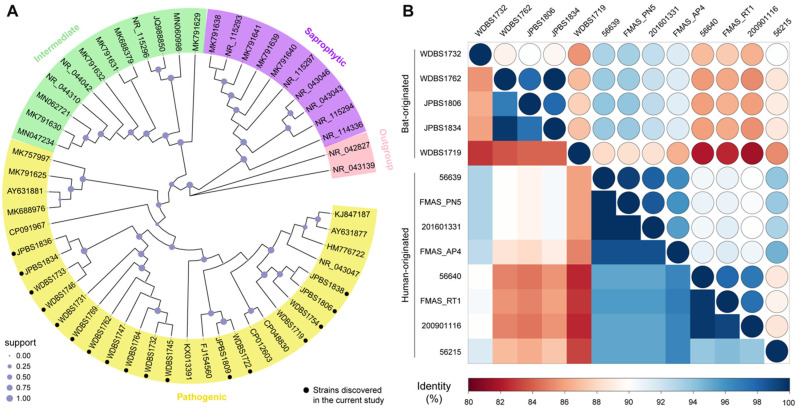
Phylogeny of *Leptospira* based on 16s RNA (*rrs*) gene and homology analysis based on the *LipL32* gene. (**A**) The phylogeny of *Leptospira*. The ML tree was reconstructed based on the *rrs* gene of pathogenic, intermediate, and saprophytic groups of the genus. The three major groups were indicated by different colors: pathogenic group (yellow), intermediates (green), saprophytic group (purple), *Turneriella parva* and *Leptonema illini* were included as the outgroup and indicated with pink. (**B**) Homology analysis of the representative novel strains and human-origin strains. The heatmap shows the percentage of nucleotide (right upper) and amino acid (bottom left) identity between the strains sequenced in this study and those of human origin based on the *LipL32* gene.

According to the nucleotide BLAST analysis of approximately 1100 bp of *rrs* fragment sequences in the GenBank, those we obtained exhibited the highest homology with *L. kirschneri* (99.07–99.25%) and *L. noguchii* (98.69%) (Table 1). The phylogenetic analysis comparing with representative strains of the genus *Leptospira* based on the *rrs* gene showed the same close relationship, which was grouped into two large branches with other validated species within the pathogenic clade (Figure 2A).

The *LipL32* gene was selected for a homology analysis because of its unique and essential role in pathogenic *Leptospira*. Comparison of both nucleotide (683 nt) and amino acid (227 aa) between the novel strain and those originated from humans revealed high similarity with *Leptospira kirschneri* (FMAS_PN5, 88.1–94.0%) and *Leptospira interrogans* (56639, 87.9–94.0%), especially strain WDBS1732 (Figure 2B). Therefore, the pathogenicity of the novel bat-originated strains discovered in this study remains unclear.

#### Multilocus Sequence Analysis

The genes *LipL32*, *flaB*, and *secY* were considered for the analysis because of the abundance of the available published sequences in GenBank. Different parameters analyzed of the three loci for 49 pathogenic strains show that the discriminatory power (DP) ranges from 0.983 (*flaB*) to 0.994 (*secY*), with the concatenated loci having the highest DP of 0.996. The number of alleles and polymorphisms reveal similar results (Table 2). Besides, considering the evolutionary ratio could interfere in the subsequent phylogenetic analysis, the ratios of non-synonymous (dN) to synonymous (dS) substitutions per nucleotide site for the tree genes were evaluated. As expected, the dN/dS was, in all cases, less than 1 (0.05986, 0.01117, and 0.01419 for *LipL32*, *flaB*, and *secY*, respectively). Therefore, we decided to perform an MLST analysis based on the three loci mentioned above to characterize the newly obtained spirochetes sequences in this study at a higher level of discrimination.

**Figure 3:**
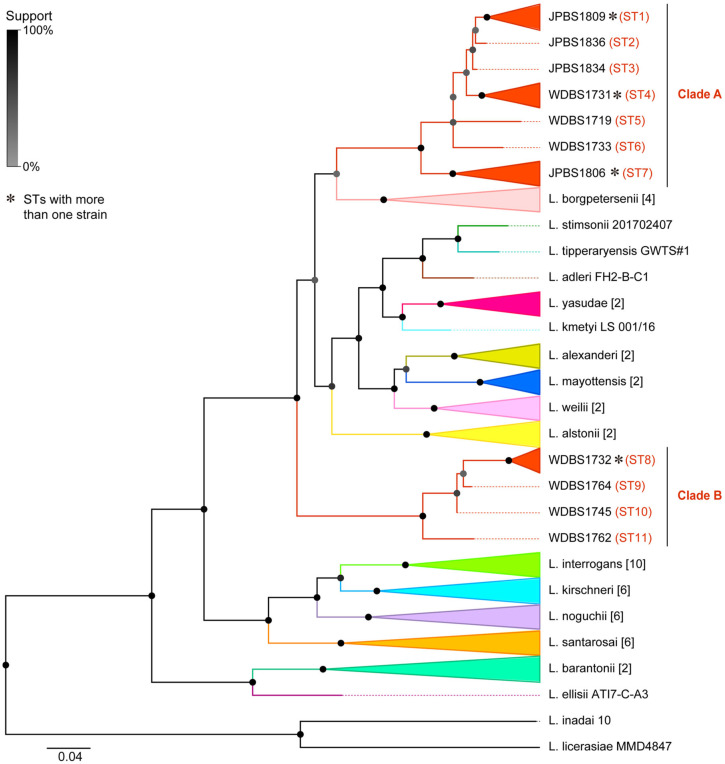
Maximum likelihood tree of the pathogenic group of genus *Leptospira* based on three loci (*secY*, *LipL32,* and *flaB*) using the MLSA method. Different colors indicate different *Leptospira* species. The number of strains in each species clade is shown in brackets. Sequence types (STs) were assigned by MLSTest and signed within the tree after the names of novel strains. The asterisks indicate STs with more than one strains (ST1: JP1809, WD1722, WD1754; ST4: WD1731, WD1769; ST7: JP1706, JP1838; ST8: WD1732, WD1747, WD1746). *L. inadai* and *L. licerasiae* of the intermediate group are used for the outgroup.

The size of the fragments analyzed in this study was 720 bp, 730 bp, and 1100 bp for *LipL32*, *flaB,* and *secY*, respectively, and resulted in a total length of 2904 bp concatenated sequences. The maximum likelihood (ML) phylogenetic tree was reconstructed from concatenated sequences of the 51 isolates and 17 newly detected strains in this study. ML tree showed 16 known clusters that matched all pathogenic species assignments, and all new sequences detected in this study rendered 11 different sequence types (STs). All new strains belong to the pathogenic group and cluster into two branches (Figure 3). In addition, the two branches obtained from bat kidneys in this study are genetically distinct from known pathogenic *Leptospira* spp., implying the discovery of two new species with potential pathogenicity. Furthermore, the strains of clade A were obtained from two locations, while clade B was found only in WD, suggesting that the species of *Leptospira* seems not clustered by geographic regions (Figure 3).

## 4. Discussion

Leptospirosis has been identified as a zoonotic disease that causes serious public health problems and severe economic losses worldwide [1,2,3,4]. It is one of the most common but neglected tropical diseases in China, as well as Yunnan Province [5,6,7,8]. Many studies have suggested that bats can be the natural reservoirs of pathogenic leptospires, and the bacteria even spill over from bats to humans and other animals [17,18,19,20,21,22,23,24,25,26,27,28]. Notably, to the best of our best knowledge, this is the first report on the molecular detection and characterization of pathogenic *Leptospira* species in bats in this region.

Although a total of 276 bats (117 frugivorous bats and 159 insectivorous bats) belonging to ten species were included in the screening, only one fruit-feeding species (*Rousettus leschenaultii*) was found to harbor pathogenic spirochetes. This finding suggests that *R. leschenaultii* was infected with pathogenic spirochetes with a frequency of 16.04% (Table S4), emphasizing the previous view that bats serve as potential natural reservoirs in circulating leptospires [57,58]. Notably, a similar prevalence of leptospires among bat populations has been reported in Papua New Guinea, Grenada, and Australia [22,32,59]. Accordingly, we suspected the high degree of habitat overlap between rodents and bats, and, thus, the shared resources between species bring individuals closer together, exacerbating the potential for spirochetes transmission [23,60]. Therefore, insectivorous bats are at a lower exposure risk than frugivorous bats who share food with rodents [61]. Nevertheless, no insectivorous bats were collected from the same sites with spirochetes-positive frugivorous bats; moreover, the habitats of the bats could not be clearly assessed, making it difficult to determine the cross-species transmission among bats.

These detection-positive bats were captured from orchards (WD) and caves (JP), closely associated with anthropogenic activities. Pathogenic spirochetes harbored by frugivorous bats may fall on fruits and/or the surrounding environment with their hosts’ activities in orchards; hence, the pathogens thereby would be exposed to humans, increasing the chance of bat-borne spirochetes spillover [61]. Besides, with the expansion of human activities and exploitation of natural resources, the opportunities for contact between bats and humans in the wild have significantly increased, which increases the risk of humans to direct or indirect contact with contaminated excreta and secretions of bats and other animals. Therefore, further investigation of the prevalence of leptospires in bats and other animals and epidemiological studies of local populations are needed. All newly detected spirochetes from bats clustered into two clades within the pathogenic group and distinct from the previously reported strains (Figure 3); therefore, we propose that these strains may represent two novel *Leptospira* species. Strains of clade A were found harbored by bats in WD and JP, while those of clade B were only circulating in the former, suggesting that the species of genus *Leptospira* in this study seems not strongly associated with geographic regions or may be associated with bats’ vagility. Previously, variants of zoonotic species have been reported in various hosts in Yunnan, including *L. interrogans* in rodents, *L. borgpetersenii* in shrew/pig, and *L. alexanderi* in cattle [62]. However, species of captured bats in spirochetes-positive sites (WD and JP) were limited, resulting in a lack of knowledge relative to other bat species. Above all, we speculate that the diversity of the genus *Leptospira* in bats is still underexplored.

The gene *LipL32* (also known as Hap1) encodes the major outer membrane protein with high immunogenicity [51]. The restriction of this protein to pathogenic serovars strongly suggests its critical role in the infection and pathogenicity of pathogenic spirochetes [50,51,52,53]. Based on the limited-length sequences, we performed homology analyses for nucleotide and amino acid sequences of the novel bat-borne and previously human-associated strains. Accordingly, the findings revealed that the novel strains were closely related to *L. kirschneri* (FMAS_PN5) and *L. interrogans* (56639) based on nucleotide analysis; however, the amino acid identity of the two (<90%) was lower than those between the validated zoonotic *Leptospira* species (>94%), except strain WDBS1732 (Figure 2B). Therefore, the pathogenicity of the novel bat-borne strains remains unclear, and further analysis is also required to elucidate protein in biology fully.

In summary, the findings from this study demonstrate diverse *Leptospira* species harbored by bats in Yunnan. However, there are some limitations revealed in this preliminary investigation. Firstly, limited bat species in the same collected site hindered a comprehensive understanding of the prevalence of leptospires in the whole bat population. Secondly, the other animals, especially rodents, were not included in the study, making us unable to explore the potential transmission route between common reservoirs of leptospires. Lastly, the prevalence of leptospirosis in the surrounding populations was not clear, and the pathogenesis of the novel bat-borne strains was not fully understood. In any case, infected bats inhabiting locations closely associated with humans always pose zoonotic threats to public health.

## Figures and Tables

**Table 1:** Result of *rrs* sequence BLAST in GenBank.

NO.	Bats Samples	Bats Species	*Leptospira* Species	Sequence Reference	Identity (%)
1	WDBS1719	*Rousettus leschenaultii*	*Leptospira borgpetersenii*	HM776722	98.6
2	WDBS1754	*Rousettus leschenaultii*	*Leptospira borgpetersenii*	NZ_CP047520	98.51
3	JPBS1806	*Rousettus leschenaultii*	*Leptospira borgpetersenii*	NZ_CP047520	98.51
4	JPBS1838	*Rousettus leschenaultii*	*Leptospira borgpetersenii*	NZ_CP047520	98.51
5	WDBS1732	*Rousettus leschenaultii*	*Leptospira interrogans*	NZ_CP048830	98.41
6	WDBS1745	*Rousettus leschenaultii*	*Leptospira interrogans*	NZ_CP048830	98.51
7	WDBS1764	*Rousettus leschenaultii*	*Leptospira interrogans*	NZ_CP048830	98.51
8	WDBS1722	*Rousettus leschenaultii*	*Leptospira kirschneri*	FJ154560	99.07
9	WDBS1731	*Rousettus leschenaultii*	*Leptospira kirschneri*	KX013391	99.25
10	WDBS1733	*Rousettus leschenaultii*	*Leptospira kirschneri*	KX013391	99.07
11	WDBS1746	*Rousettus leschenaultii*	*Leptospira kirschneri*	KX013391	99.07
12	JPBS1809	*Rousettus leschenaultii*	*Leptospira kirschneri*	FJ154560	99.07
13	JPBS1834	*Rousettus leschenaultii*	*Leptospira kirschneri*	KX013391	99.07
14	JPBS1836	*Rousettus leschenaultii*	*Leptospira kirschneri*	KX013391	99.07
15	WDBS1747	*Rousettus leschenaultii*	*Leptospira noguchii*	NZ_CP091967	98.69
16	WDBS1762	*Rousettus leschenaultii*	*Leptospira noguchii*	NZ_CP091967	98.69
17	WDBS1769	*Rousettus leschenaultii*	*Leptospira noguchii*	NZ_CP091967	98.69

**Table 2:** Parameters which were analyzed in a subset of 49 isolates of the pathogenic cluster.

Parameters/Loci	*secY*	*LipL32*	*flaB*	Concatenated Loci
Number of alleles	42	36	33	44
Number of polymorphisms	448	193	362	1003
Typing efficiency	0.094	0.187	0.091	0.111
DP(95% confidence interval)	0.994(0.989–1)	0.986(0.974–0.997)	0.983(0.973–0.993)	0.996(0.991–1)
dN/dS	0.01419	0.05986	0.01117	0.03877

## Data Availability

The sequences obtained in this study have been submitted to GenBank with accession numbers OQ581805-OQ581821 (*rrs*), OQ599467-OQ599438 (*secY*), OQ599484-OQ599500 (*flaB*), and OQ599501-OQ599517 (*LipL32*), respectively.

## Supplementary Materials

The following supporting information can be downloaded at: https://www.mdpi.com/article/10.3390/microorganisms11061619/s1, Table S1: Primer sequences for the amplification of pathogenic *Leptospira*-related genes; Table S2: Accession and information of sequences used for *rrs*; Table S3: Accession and information of sequences used for MLST; Table S4: Frequency of molecular detection of leptospirosis pathogens in bats collected in Yunnan.
